# The Use of Novel Stimulants in ADHD Self-Medication: A Mixed Methods Analysis

**DOI:** 10.3390/brainsci15030292

**Published:** 2025-03-10

**Authors:** Tayler Holborn, Fabrizio Schifano, Emma Smith, Paolo Deluca

**Affiliations:** 1 Institute of Psychiatry, Psychology & Neuroscience, King’s College London, 16 De Crespigny Park, London SE5 8AF, UK; tayler.j.holborn@kcl.ac.uk (T.H.);; 2 School of Life and Medical Sciences, University of Hertfordshire, Hatfield AL10 9AB, UK

**Keywords:** novel psychoactive substances (NPSs), self-medication, ADHD, stimulants, 2-FMA, 4F-MPH

## Abstract

Background: Numerous individuals suffer from attentional issues, such as ADHD. While medication is considered the first-line treatment, it is unavailable to some. As a result, certain individuals are choosing to self-medicate with novel stimulants, a phenomenon that remains poorly understood. We aimed to investigate which NPSs are being used to self-medicate ADHD, evaluate their perceived effectiveness, and explore the experiences and motivations of those self-medicating. Methods: Data from respondents (*n* = 225) (mean age [SD] = 29.5 ± 9.6; male = 83%; female = 12%; non-binary = 5%) were collected via an online survey, with nine participants (mean age = 31.4; male = 5; female = 1; non-binary = 3) undertaking further semi-structured interviews and the data being investigated using a framework analysis. Results: The most-used NPSs were 4F-MPH and 2-FMA. Some individuals perceived self-medication to be more effective than conventional treatment (*p* < 0.001). A framework analysis identified the following themes surrounding novel stimulant self-medication: (1) the use of NPS stimulants as a stopgap between treatments; (2) poor access to ADHD treatment; (3) a lack of openness and confidence in psychiatrists and healthcare providers. Conclusion: Novel stimulants are being used when access to ADHD treatment is poor. Interventions should aim to reduce long treatment wait times and issues surrounding geographical access. Careful consideration should be given before denying stimulant medication to individuals with co-occurring substance use and psychiatric comorbidity. Individuals desire a more patient-centred ADHD treatment with broader pharmacotherapies.

## 1. Introduction

Attention deficit/hyperactivity disorder (ADHD) is a neurodevelopmental disorder defined as “a persistent patten of inattention and/or hyperactivity and impulsivity that interferes with functioning or development”, with three recognised subtypes: a hyperactive–impulsive type (ADHD-PH), an inattentive type (ADHD-PI), and a combined type (ADHD-C) [1]. Originally classed as a purely developmental disorder, prevalence rates for ADHD in children are approximately 4 to 7% [2]. Early research focused on the association with school-age boys (4 to 17), where survey data indicated them to be 2.5 times more likely than girls to be diagnosed with ADHD [2]. More recently, the notion of adult ADHD is being considered [3]. Despite controversy, data suggest that adult ADHD is underdiagnosed [4] and estimated to affect 2.5% of adults worldwide [5], persisting into adulthood in around two-thirds of childhood cases [6,7,8]. Diagnoses for both adults and children appear to be increasing [9], and there is growing awareness of the significant social and economic impairment felt by many adults [10], especially given its high comorbidity with other psychiatric disorders [11].

Current treatment focuses on pharmacotherapy with stimulants and, less often, non-stimulants, usually in conjunction with non-pharmacological treatment [12]. These treatments are largely accepted worldwide for adolescents; however, there is less consensus on the management of adult ADHD [13]. In 2018, the National Institute for Health and Care Excellence (NICE) updated its guidance for ADHD [14], outlining methylphenidate (MPH) as the first-line treatment for children, adolescents, and adults. Adults can also be offered lisdexamfetamine (LDX), with research indicating that amphetamines may be more efficacious in adults than children [15]. Despite guidelines, international differences in prescribing patterns remain, especially regarding adult treatment [16], and the time taken to receive a diagnosis can vary considerably [17]. Finally, due to its consideration as a developmental disorder, there is still a need to validate the experience of ADHD symptoms in childhood. It is likely this requirement is feeding into the underdiagnosis of ADHD in adults [17].

Recently, there have been global shortages of ADHD medication [18,19] due to increasing diagnoses and prescriptions, exacerbated by the impact of the COVID-19 pandemic and global supply issues. Instances of self-medicating are evident [20], with data suggesting that inadequate insurance or difficulties obtaining medication as an adult are factors which lead individuals to seek alternatives to prescription stimulants [20].

Self-medication, defined as “choosing and using substances to address self-diagnosed symptoms without consulting a doctor”, can afford an individual benefits but also poses considerable dangers such as a higher risk of adverse effects, a prolonged duration of use, misdiagnosis, and the use of excessive or incorrect dosages [21]. Social, cultural, and economic factors, such as income, have been shown to influence self-medication [22]. Self-medication is practiced almost ubiquitously, including the use of over-the-counter (OTC) medication [23]. Data suggest self-medication is often higher in younger populations, such as students, [24] and, historically, has been advanced as a general theory of substance use [25,26], with strong links evident between ADHD and illicit substance use [27]. Studies have demonstrated that those suffering from ADHD were more likely to use cocaine, non-prescription stimulants [28], and amphetamines [29]. Data also indicate that nicotine dependence in patients could largely be explained by self-medication [30], and, recently, qualitative research has shown that individuals are using Novel Psychoactive Substances (NPSs) to self-medicate ADHD [20].

NPSs, also referred to as “new” psychoactive substances, “legal highs”, or “research chemicals”, are a heterogenous and transient category of substances, defined as “substances of abuse, either in a pure form or a preparation, that are not controlled by the 1961 Single Convention on Narcotic Drugs or the 1971 Convention on Psychotropic Substances, but which may pose a public health threat” [31]. NPSs often mimic classic illicit substances, such as cocaine [32], or regulated substances, such as methylphenidate [33], with the category consisting of a variety of substances not limited to opioids, psychedelics, synthetic cannabinoids, and benzodiazepines. Whilst including “novel” or “new” in their syntax, they may not be comprehensively new inventions but instead may be substances that have recently appeared on the drug market, sometimes after previously being an unsuccessful pharmaceutical. This broad definition of NPSs has attracted criticism [34]; however, the proliferation of NPSs remains a consideration for authorities [35], and their online availability and grey area legal status make them an appealing option for self-medication. Previous data highlight the use of novel stimulants that are structurally related to ADHD medication, including *2-fluoromethamphetamine* (2-FMA) and *4-fluoromethamphetamine* (4-FMA) [20]. The aims of the current study are to determine an up-to-date account of the NPSs used to self-medicate ADHD, evaluate their perceived effectiveness and the influence of income, and explore the experiences and motivations of those choosing to self-medicate ADHD.

## 2. Materials and Methods

A cross-sectional online convenience survey was launched using Qualtrics between October 2023 and July 2024. To be eligible, participants had to be ≥18 years old. The participants were recruited using advertisements on NPS-related websites [20,36], including Bluelight.org, Drugs-forum.co.uk, partyvibe.org, and Reddit (https://www.reddit.com/r/researchchemicals, accessed on 1 July 2024). Following this, semi-structured interviews were completed between August and October 2024 with nine participants, and the data were examined using a framework analysis.

The survey’s demographic questions included the participants’ gender, age, ethnicity, country of origin, highest level of education, income, and history of drug use. The participants were asked which disorder they suffered from and whether their disorder was diagnosed by a medical professional. A free text box was available, and the participants were able to declare up to two disorders, where they could rate the effectiveness of conventional treatment and NPS self-medication for each, repeating a section of the survey. The participants were then asked to rate the perceived effectiveness of conventional treatment and NPS self-medication using a 100-point slider scale. When declaring which NPSs were used, the participants were presented with a list of 28 NPSs implicated in the self-medication of ADHD, as identified in previous research [20,37], as well as a free text box. There were no limits on the number of NPSs they could select for each declared disorder. In the post-hoc analysis, any answers that mentioned generic category terms, such as “amphetamines”, were discounted. Finally, the participants were asked to rate their perception of conventional healthcare using a 100-point slider scale and declare whether their NPS use or diagnosis came first. A copy of the survey is available (Supplementary Material S1).

The participants were contacted following survey completion and offered the opportunity to be interviewed. Twenty-two of the participants responded and consented to participate. Nine of the participants completed the interview. Prior to the data collection, a semi-structured interview guide was constructed focusing on four topics: (1) the history of NPS use; (2) views on NPSs; (3) self-medication practices; (4) perceptions of conventional healthcare. A pilot interview was undertaken to ensure interview comprehension and validity. Saturation was estimated, based on previous research, to be around 15 [38,39,40]; however, no new codes were introduced after eight interviews, indicating saturation [41]. Following the interview process, all identifiable information was discarded, and the participants’ data were discussed only in relation to their identification codes. The interviews were recorded using Microsoft Teams and transcribed using KCL software, with the data generated coded using NVivo 14 [42] and Microsoft Excel [43]. A copy of the semi-structured interview guide (Supplementary Material S2) and emergent codetree are included (Supplementary Material S3).

A framework analysis was used to analyse the interview data [44,45], affording an initial deductive interview guide, with the opportunity to update iteratively as we progressed. In line with Gale et al. (2013) [44], we first transcribed all the audio recordings, making notes in the margin of the transcripts. This was done whilst undertaking interviews, allowing for themes to be revisited as further interviews progressed. After completing the data collection, the audio recordings were re-listened to and notes on overarching themes were made. Transcripts were coded line-by-line using NVivo 14 [42]. Our original codetree, informed by previous research [20], was expanded as the coding progressed. Once a working analytical framework was developed and each transcript coded, the data were charted into framework matrices pertaining to the emerging themes. The framework matrices were then exported into Microsoft Excel, allowing for qualitative data to be mapped, summarised, and interpreted for discussion. The framework matrix data are available upon request.

All the data were processed using SPSS 27.1 [46], R studio [47], and NVivo 14 [42]. For the survey data, frequencies are reported for the demographic information. Means (±SD) are given for ages and for the self-rated effectiveness of conventional treatment and NPS self-medication, and paired-sample T-Tests were used to compare the means. An ANOVA was used to investigate the effect of income.

## 3. Results

### 3.1. Survey Results

#### 3.1.1. Sample

In total, 380 individuals started the survey. The participants were excluded for not finishing the survey (*n* = 116) and for not using any NPSs to self-medicate (*n* = 39). Overall, 225 individuals completed the survey, were older than 18, and were eligible for analysis. The median survey completion time was 5 min 54 s.

The respondents were predominately male (82.7%), with 26 female (11.5%) and 11 non-binary (4.9%) participants. The participants’ age ranges (mean age [SD] = 29.5 ± 8.6) consisted of 18–25 (*n* = 95), 26–35 (*n* = 107), 36–45 (*n* = 34), and 45 + (*n* = 14). They were mostly white (86.7%) and predominately from the USA (25.7%), the Netherlands (12.8%), and Germany (10.2%). Most of the respondents (23.0%) earned between USD 25,000 and USD 49,999 and had achieved a high-school education (40.3%). The most commonly used non-NPS substances were alcohol (82.2%), cannabis (75.3%), and nicotine (72.7%).

#### 3.1.2. Disorders

Two hundred individuals declared themselves to suffer from one disorder and twenty-five individuals declared themselves to suffer from two disorders, resulting in two hundred and fifty overall declared disorders (Table 1). The majority of the participants declared themselves to have been diagnosed by a medical professional (76.1%), with most (71.0%) having been diagnosed before initiating NPS use.

#### 3.1.3. Treatment, Self-Medication, and Healthcare Perceptions

Over a third (34.0%) had received both therapy and medication. A similar number (33.6%) had received medication. Some 10.4% had received only therapy, and 18.4% had received no treatment. The ratings of the perceived effectiveness of conventional treatment and self-medication, as well as the perceptions of healthcare, are included below (Table 2).

#### 3.1.4. NPSs Used

When looking to self-medicate, the most used NPSs were 4F-MPH, 2-FMA, isopropylphenidate, 2-FA, and 3-FPM (Table 3).

#### 3.1.5. Income and Self-Medication

**Figure 1:**
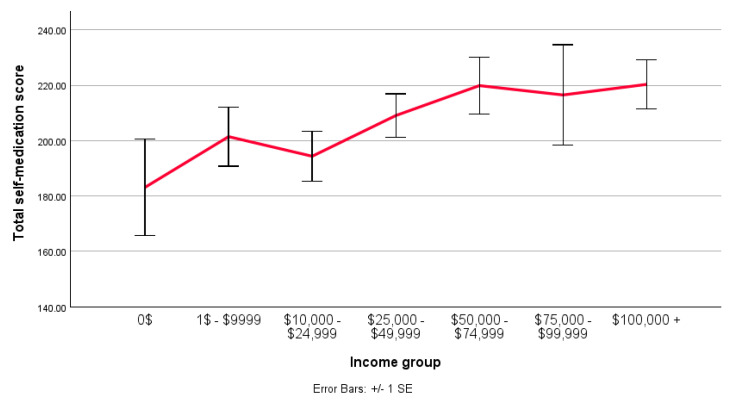
A line graph comparing the mean total score (0–300) for the perceived effectiveness of NPS self-medication across different income levels. Made in SPSS.

The scores for each measure (“Did it work”, “Did symptoms disappear”, and “Did QoL increase?”) were combined, and the groups were split by income level (Figure 1). A regression analysis was conducted, with the model’s constant indicating a baseline effect (B = 183.125; SE = 15.265; t = 11.996; *p* < 0.001). None of the individual income groups indicated statistical significance (*p* > 0.05). However, two groups approached significance (“$50,000 to $74,999”, *p* = 0.64; “$100,000+”, *p* = 0.65).

### 3.2. Interview Results

The eligible participants were interviewed until the saturation of themes was reached, and, therefore, no further advertisement for sampling was conducted. The interviews lasted for an average of 38.9 min. The participants were male (*n* = 5), female (*n* = 1), and non-binary (*n* = 3). The locations ranged between Bulgaria, France, Germany, Greece, Netherlands, United Kingdom, United States, and Spain, with the ages ranging from 20 to 60 (mean age = 31.4). All the participants demonstrated some disorder comorbidity, including depression, anxiety, post-traumatic stress disorder (PTSD), anhedonia, trauma, narcolepsy, autism spectrum disorder (ASD), chronic fatigue, and suicidal ideation. The timelines of self-medication varied from 3 months to 14 years. The interviews revealed the following themes surrounding NPS use: (1) NPS stimulants used as a stopgap between treatments or as a supplement; (2) poor access to ADHD treatment; (3) finding a “good” psychiatrist; (4) a lack of openness and confidence in healthcare providers. Evidence is provided in the form of quotes taken from the themes analysed via a framework analysis. Additional sample quotes are provided (S4).

#### 3.2.1. Initiating NPS Use

The participants outlined similarities in their first use of stimulants, including both novel and prescription use. This initial use was prompted by a feeling of being unable to “keep up” with peers, usually in an academic or occupational setting. Issues with social interactions were also mentioned. These events often engendered the individual to consider a diagnosis of ADHD.

“*In university or at school for exams, like my mind would feel by itself [even] without any diagnosis that I need amphetamines to pass the exams, because if not then I will be just very, very slow in completing them.*”

*F33*

Exposure to the NPS market was usually through an online means, with individuals expressing a desire to avoid stimulants from the illicit market. The participants would often describe finding out about NPSs through the internet, including through forum-mediated discussion, such as via Reddit.

“*And at school, there were some stimulants, variable quality. And I didn’t want to deal with them. And so I was looking for anything “legal”, which could be imported without any problems*…”

*M38*

“*At the time when I was a student and when I was feeling that this is becoming to be a problem for me, not following my peers and not understanding what the teacher is talking about in class, it was a problem, and motivation to finish things and many things associated with ADHD. I read about it on the internet and I kind of self-diagnosed and started to self-treat*”

*M38*

“*First time I read about Modafinil on Reddit. I’m not a big Reddit user […] but I read about Modafinil there for the first time. And then I read about 2FMA there too about people who are using it, and they worked for them etc. And I risked by trying it*…”

*M60*

The participants had significant comorbidities, including depression, anxiety, PTSD, anhedonia, trauma, narcolepsy, Aspergers, chronic fatigue, and suicidal ideation. Eight of nine participants at the time of interview had an ADHD diagnosis and were using, or had previously used, prescription stimulant medication. Comorbid disorders tended to be chronic, and individuals had not found efficacy in therapy or other medication. Often, an ADHD self-diagnosis was initially made through reading online testimonies before approaching a healthcare provider.

“*Yeah, so I think it’s been a while that I’ve been suspecting ADHD because I’ve read testimonies online, which I felt were close to what I was feeling. And I also checked Wikipedia, […] which did correspond to my situation*.”

*M27*

Novel stimulants were used either as a stopgap before or in between prescription medications. They were also used as a supplement to an ongoing prescription that was perceived to be insufficient due to a maxed out legal dosage or perceived limitations with extended release (XR). Some countries favoured non-stimulant medications, and this caused some of the participants to pursue novel stimulants. Concurrently, non-stimulant NPSs were also being used to self-medicate comorbid disorders, including depression and anxiety.

“*I already had an issue with the dosage of it [Ritalin] because for some reason, I have a high natural tolerance to where the maximum legal prescription where I live in France is 80 milligrams, which is not high enough for me from the beginning*…”

*N24*

“*I was having trouble obtaining prescriptions that I found useful to me for some various mental health stuff I had going on at the time. So decided to look around elsewhere and found a pretty accessible market in the research chemical spaces*”

*N20*

For those initiating self-medication, limited access to treatment was a key issue. Long wait times to initially see a psychiatrist were outlined by all the participants (3 months to 2 years). For the participants not living in their native country, such as international students, there were often difficulties in finding the appropriate route to gain a prescription, as well as confusion over whether they would be eligible for insurance coverage or whether their prescription would transfer back home after studying. The participants also outlined issues regarding geographical access when living in remote areas.

“*Yes, the access is very limited. And then […] for Berlin, for example, or Hamburg, I am like 300 km from there, so that’s a three-hour ride, no matter with what, if by car or on the train or whatever, it’s too far, so yeah, it’s pretty complicated*.”

*M38*

“*I have a lot of friends […] trying to get diagnosed in the UK in general and a lot of them are on like year-long waiting lists for the screening*”

*M21*

In contrast, NPS access was deemed reasonably easy, especially in the Netherlands, although had become more difficult following changes in legislation and the removal of normal card payments. Whilst the participants were reticent to fully disclose where exactly they obtained their NPSs from, it was clear that most utilised the internet and sometimes international delivery if not based in the Netherlands; however, this was becoming more difficult. One individual also described using the Darkweb (S4).

“*Since then I’ve used 3FMA and 4FMA, and 4FMA is a bit different. Nowadays 2FMA became very difficult to find because it became illegal in most countries*.”

*M60*

Other motivations for self-medicating with novel stimulants included the financial cost of ADHD treatment and medication, as well as the issues of medication shortages. One participant described how self-medicating afforded increased privacy from his family.

“*I just was not super interested in like going to see a therapist or going to see a doctor to get a prescription, which my parents are going to have to, you know, put through to the insurance and they’re going to see all this stuff*.”

*M21*

#### 3.2.2. Finding the “Right” Psychiatrist

The participants would consistently describe how they had been lucky to find a “good” psychiatrist or how they were attempting to find one. Upon investigation, this description of a “good” psychiatrist referred to one that needed less convincing of adult ADHD, was more likely to prescribe stimulants, or was more open to listening to their needs.

“*I think I might have just gotten lucky with the psychiatrist I have because he kind of accepted it at face value when I told him I had those suspicions […] that I might have ADHD, and from then on the process of getting diagnosed was actually surprisingly easy*.”

*M21*

“*I had the luck that I found my psychiatrist which is able to prescribe me some proper medication, amphetamines*...”

*M60*

#### 3.2.3. Self-Medicating

Individuals were aiming to self-medicate ADHD with substances including 2-FMA, 4F-MPH, isopropylphenidate, 3-CMC, 3FPM, and modafinil. These were used in a similar way to prescription stimulant medication, with doses being taken in the morning to take advantage of the stimulant properties during working hours. The most used was 2-FMA, with dosages varying from 10 mg to 50 mg:

“*I usually take about 10 to 15 milligrams in the morning and then that’s it for the day. It does help for motivation and it’s also easier for me to keep focus on something for a longer time*.”

*M27*

“*I’m using 2FMA for like Monday, Wednesday and maybe Friday, a strong dose that’s like 45 milligrams, that’s one and a half pills once around 11 a.m*.”

*M60*

“*I would take like 50 milligrams at most. I mean, throughout the day once or twice, three times at most, but I avoided using this in the evening because then you wouldn’t sleep well*”

*M36*

Comorbid disorders were also being self-medicated, with the participants describing the use of 2-CB, LSA, and 2-FDCK for depression, as well as bromazolam for anxiety. Often, this use was based on an existing research protocol. Some had considered novel treatments, such as legal ketamine treatment or a psychedelic retreat; however, these were always deemed too expensive. One participant outlined his desire to replicate MDMA therapy using the novel substance mix named “Borax”, which involved the empathogenic phenethylamine 6-APB. Further, there was a dislike of antidepressant medications, with many outlining issues with side effects causing the cessation of use.

“*I would like to get my fingers on 6APB for the same reason as MDMA. But yeah, it’s not possible to get any more probably without violating any laws*.”

*M38*

Overall, NPSs were perceived as being of good quality, although there was still an acknowledgement of the risk given the lack of oversight from a healthcare professional. There were instances of individuals attempting to test their NPSs; however, this was sometimes deemed difficult, with some tests not being sensitive enough to determine between substances.

“*So for the most part, I think the quality was pretty good. I tried to test a lot of the stuff that I got, and for the most part didn’t really have issues*”

#### 3.2.4. Side Effects

Side effects during stimulant self-medication were evident, including paranoia, exhaustion, insomnia, and lymphatic node inflammation. Individuals chose not to use them on a regular basis due to concern over side effects, although others described NPS stimulants as having fewer side effects than their prescription counterparts.

“*I don’t use it [3-FPM] because I am afraid of the side effects, so actually I don’t use them on the regular basis, I use them mostly if I know the start of the day will be hard*.”

*M38*

“*In the past like I had been put on Ritalin and I found that to be like absolutely full of side effects whereas like 2fma was just a very clean just like not a lot of side effects*.”

*M21*

#### 3.2.5. Healthcare Perceptions

Many of the participants shared the feeling that psychiatrists lacked the knowledge needed to treat them properly, did not understand them or their needs, and often displayed stigma. One participant described how ADHD was sometimes not recognised by professionals in their country and how it was perceived to be over-medicalised by Western countries. Often individuals described feeling as though doctors were following a script instead of responding to them as an individual. Some believed the side effects of prescription medications were not outlined clearly before prescribing.

“*I was thinking that that psychiatrist... she’s just an idiot and that I know about ADHD much more than she does*.”

*F33*

“*The overall attitude towards ADHD [in Bulgaria] is still kind of something coming from The West, which is actually not an illness, but something normal, which happens to a lot of children, and it’s not something that should be treated*.”

*M38*

“*I think overall the mental health care here does not know nearly as much about these conditions and these drugs as they are suggesting that they do*”

*N20*

“*It is a lot of just: a patient comes into the office, and you throw different medications at them until they stop complaining. And I think that that’s not really the most responsible way to approach things, especially when a lot of these medications […] have a lot more side effects than is let on*”

*N24*

All the participants were under the impression that being open with a psychiatrist regarding their drug use would hinder them in obtaining adequate prescription medication in the future and, therefore, did not share their NPS use. One participant was open with their substance use and described how the psychiatrist was unwilling to prescribe stimulants following this, even after a period without stimulant use.

#### 3.2.6. Reflections

When asked to reflect on their period of self-medication with novel stimulants, most described a positive experience.

“*I wish I had discovered them way earlier, because I lost so many jobs, so many lost opportunities, so many problems, so much suffering. For me it was hell on earth to do anything. […] It’s not like I don’t want to work. It’s not like I’m not good at my work… Nobody was believing me*.”

*M60*

Despite this, at the point of undertaking the interviews, most of the participants had ceased regular NPS use, with one the participant testing a prolonged period of sobriety for health benefits. It was clear that the participants would rather be on prescription medication and did not want to be on the wrong side of the law. One individual highlighted that using prescription medication carried less social stigma and was easier to discuss with friends and family, and this was a motivation to cease NPS use.

“*If I could, instead of supplementing the prescription I have with NPSs, […] if I just got a better prescription than for sure, I would take it [instead]*.”

*N24*

“*I didn’t want to be a criminal. I wanted to medicate myself*.”

*M36*

The participants described how tolerance can become an issue with self-medication and routinely expressed how discipline and control are required for effective self-medication. One individual expressed stress at the prospect of running out of 2-FMA. Finally, one participant indicated the value of a good circle social in preventing negative outcomes.

“*I have a little bit of stress, you know, because at some point when I run out of 2FMA I have to replace it with something and right now I don’t have a good alternative*...”

*M60*

“*If you have like a healthy social circle and like things to look forward to, […] it’s much more attainable to do it [self-medication] responsibly, have people catch you on your downfalls, and like it’s [self-medication] definitely net positive, at least in my experience, definitely net positive*.”

*M21*

Overall, the perceptions of conventional healthcare were substantially negative. The participants described wanting a more cooperative, patient focused healthcare experience, with more options when it came to medication. They also described wanting more humility from doctors when it came to the knowledge of psychiatric disorders.

“*I wish that there was more cooperation between the doctor and the patient to determine a good medication regimen for them. Like, I did not appreciate getting put on medications that I didn’t find too helpful, but had a lot of side effects*”

*N20*

## 4. Discussion

We conducted, to our knowledge, the first study exploring the self-medication of ADHD with Novel Psychoactive Substances (NPSs). By means of an online survey, supplemented with a qualitative analysis, we aimed to demonstrate the NPSs being most used to self-medicate, examine their perceived effectiveness, and explore the motivations and experiences of those self-medicating. We confirmed 2-FMA and 4F-MPH as common choices for self-medicating ADHD, and we outlined that the main motivations for seeking NPSs stimulants are as a stopgap before or between treatments or to supplement an existing prescription medication.

The survey data indicated that most (89.6%) of the participants were suffering from either attention deficit disorder with hyperactivity (ADHD) (F90.0) or attention deficit disorder without hyperactivity (ADD) (F98.8). It is worth noting that subtle differences exist between the DSM-5 and ICD 10 diagnostic systems, although these will become more aligned with the introduction of the ICD-11 [48]. More broadly, research has questioned the existence of distinct subtypes [49], especially in adult ADHD, where comorbid psychiatric disorders can obscure symptoms [50]. Given the sample size and type, the current findings should be considered in the wider context of attention deficit disorders and not focused on a specific subtype.

Unsurprisingly, the most used NPSs for self-medication were stimulants, consisting of amphetamines (2-FMA, 2-FA), phenidates (4F-MPH, isopropylphenidate), and one case of phenmetrazine analogue (3-FPM). These findings illustrate consistency with our previous research, suggesting 2-fluoromethamphetamine (2-FMA) and 4-fluoromethylphenidate (4F-MPH) to currently be the most common choices for self-medicating ADHD [20]. The stimulant 4F-MPH was used by almost half (49.6%) of the sample. This substance is the fluorinated analogue of methylphenidate, a prescription medication for ADHD [51], which acts primarily as a reuptake inhibitor for norepinephrine and dopamine [52,53]. Several methylphenidate-related NPSs exist [54], including 4F-MPH, 4-fluoroethylphenidate (4F-EPH), and isopropylphenidate. It is thought that 4F-MPH exerts its effects similarly to methylphenidate, with limited monoamine interaction [33]. Data suggest it to be of higher potency [55], with an increased potential for abuse, due to increased dopaminergic selectivity [56,57]; however, human data are scarce. Notably, 4F-MPH and 4F-EPH have been involved in fatal intoxications [58,59].

Used by 46% of the sample, 2-FMA was the second most common choice for self-medication. First reported in 2007 [60], data on human use are limited, although recent reports highlight its involvement in chemsex [61]. It is the fluorinated analogue of methamphetamine and closely related to the prescription medication Adderall. Research has discussed potential concerns with the long-term use of fluorinated analogues, such as 4F-MPH and 2-FMA [20], although evidence in this area is lacking. Other stimulants used for self-medication included isopropylphenidate (20.4%), 2-fa (19.2%), and 3-fpm (17.2%). Our data lend support to the self-medication hypothesis [25,26], with 71% of the participants declaring their NPS use to follow their diagnosis. Similarities between prescription medication and popular NPSs for self-medication imply a degree of rational choice when self-medicating [62], opposed to an unconscious attempt to relieve trauma or suffering. Indeed, others have observed active substance substitution [63] in situations where treatment options may remain out of reach due to geographical or financial constraints.

Research highlights that drug use self-report data can be reliable [64], and specifically, data concerning e-psychonauts and NPSs have suggested that there could be low (6.2%) levels of adulteration [65]. Regardless, we make no assertions of causation given the convenience sample, and there is a possibility that individuals may have been deceived whilst purchasing, especially if this was through the Darknet or illicit means, as opposed to the Clearnet. Additionally, no attempts were made to verify the improvement of the participants with doctors, and we acknowledge the possibility of fabricated self-report data. Future research could utilise this measure to improve reliability in the data and explore the possibility of substance abuse after improvement.

The NPS users in the current study often perceived NPSs as being of good quality, and this has been described as a motivation in previous research [66]. While a limitation of the current study is the inability to confirm any of the substances discussed, it is possible that when purchased from the Clearnet, suppliers would feel some pressure to make sure their substances were accurately labelled and somewhat free from impurities as any deaths may push governments to pursue restrictive legislation that would impact their businesses. The recent introduction of blanket ban legislation and integration with the illicit substances market may, therefore, reduce this quality. With these prior limitations in mind, the current study suggests that NPSs were perceived as significantly more effective than the conventionally offered treatment across all the measures. We suggest that the perceived increased effectiveness of NPSs is more likely to reflect the stigma towards conventional healthcare [20], rather than a genuine improved efficacy. Whilst non-significant, our regression analyses suggested that a higher income was associated with the greater efficacy of self-medication. The influence of demographic and socioeconomic factors on self-medication is complex [67], with research suggesting that the male sex, a young age, and being Caucasian are factors that are linked to individuals self-medicating [68,69,70,71]. Previous data have demonstrated that self-medication is practiced by those of low and high incomes [72], providing value to both [73]. In the current study, it is possible that those with higher incomes were more likely to be part of the healthcare system and, therefore, could have used NPSs to supplement their medication. Alternatively, those on lower incomes may have looked to use NPSs *instead* of obtaining prescription medication and had not benefitted from medical oversight. Finally, as outlined by one participant, we introduce the role of peer support in mediating self-medication efficacy. The notion of peer support being associated with reduced substance use is not new [74,75], with substance use and societal norms being deeply entwined. Research focusing specifically on self-medication has suggested that both a supportive peer group [76] and parental support [77] may buffer the risk of engaging in self-medication. Our research extends this notion, suggesting that once self-medicating, a peer group could be protective against the adverse effects of self-medication.

Research has outlined differences between NPS users and other substance user populations [78]. We hypothesise that NPS users, on average, may have a higher pharmacological knowledge than regular illicit drug users and that this may cause them to be more likely to self-medicate. Indeed, the NPS users deemed *“Psychonauts”* were seen to possess a high level of knowledge [79,80], with some even suggesting they should be consulted regarding policy [81]. Similarly, the effect of knowledge has been shown in a meta-analysis of students, where the prevalence of self-medication was higher (97.2%) in medical students than non-medical students (44.7%) [24].

We highlight that a large degree of our survey sample (78.4%) reported polydrug use, and all the individuals interviewed declared a comorbid disorder. Other studies have demonstrated a high prevalence (21–23%) of substance use disorder (SUD) in adult ADHD [82,83], with the risk of psychiatric comorbidity being increased if the individual has both SUD and ADHD [84]. Comorbid ADHD sufferers are also more likely to be risk-seeking [85] and have higher levels of stimulant use [86]. Furthermore, these individuals are likely to experience increasing difficulties remaining abstinent [87] and may suffer from the reduced effectiveness of standard-dose pharmacotherapy [88]. This last point seems to resonate particularly with the current findings, given that the interviewed participants explicitly outlined their available dosages as being insufficient, and this was a motivator in their decision to supplement with novel stimulants. Correspondingly, this may explain why these individuals sought to self-medicate originally and why they perceived self-medication to be more effective than prescription stimulants, as this allowed them access to a higher dose. However, this does add complexity to clinical treatment, given the proclivity of some ADHD patients to exaggerate symptoms to obtain stimulants, and as such, thorough follow-up evaluations should be undertaken [89]. Despite the controversy surrounding the prescription of stimulants to those with ADHD and SUD, the evidence suggests that those left untreated experience worse outcomes [82], especially when compared to those treated early on [90]. Often, abstinence is a requirement for pharmacotherapy [91]; however, as outlined in our study, this can cause the patient to deceive healthcare providers, as they suspect being truthful will reduce their chances of obtaining pharmacological treatment. Overall, the evidence suggests no complications or increases in substance use with pharmacological treatment [92] and efficacy in general [93]. Therefore, clinicians should carefully consider the decision to avoid stimulant treatment in those with comorbid substance use.

In addition to psychiatric comorbidity, sleep disorders such as narcolepsy are often seen in ADHD patients [94], and this was observed in a small number of the participants in the current study. There are data suggesting that sleep issues in adults may be bidirectionally associated with ADHD severity and other comorbid disorders, such as depression, anxiety, and substances use [95]; however, this intersection, especially considering the use of NPSs in adults, is a topic that requires more research to be adequately understood.

The qualitative analysis unveiled four main themes: (1) the use of NPS stimulants as a stopgap between treatments or to supplement a prescription medication; (2) poor access to ADHD treatment, with long waits evident (3 months–2 years); (3) finding a “good” psychiatrist; (4) a lack of confidence in healthcare providers and the need for a “patient-centred” approach. Previously, data have indicated that difficulties in obtaining adult ADHD diagnoses would be the main motivator for those seeking NPSs [20]; however, the current research suggests that may be incorrect, and data from other populations confirm the utility of self-medication as a stopgap before going on to receive medical treatment [96]. Whilst broader research has shown that self-medication with non-prescription medications is common, especially among students [97,98,99], it is likely that the current sample was not motivated to attempt this. Firstly, some individuals already had a prescription available and were supplementing their use with NPSs, rending non-medical use redundant. Secondly, our qualitative data suggest that if they were trying to obtain a prescription, then NPS use was ceased once that prescription was obtained. Further, our data demonstrate an ease of access to the NPS market through internet means and a perceived higher quality in comparison to illicit substances. It is likely that these factors caused individuals to focus on NPSs as an option and not illicit stimulants or non-medical prescription medication.

Issues with treatment access were, however, evident. Data on the treatment of ADHD have shown high variation between countries [100,101], and this variation in treatment was corroborated by our qualitative research. These data confirm that much can be done to improve access in terms of wait times [102] and barriers to treatment [103]. Two interlinked themes regarding conventional healthcare emerged in the current study: the issue of finding a “good” psychiatrist and the lack of confidence in the knowledge of healthcare providers. These sentiments are echoed in research, suggesting a lack of training in practitioners [104], unhelpful attitudes regarding the construct of ADHD [105], and differences in the confidence and prescribing practices between psychiatrists and non-psychiatrists [106]. Research in the UK has recommended that adult ADHD should not be relegated to secondary or tertiary services [107], and clearly steps need to be taken globally to improve the treatment of adult ADHD. Compounding these issues was the feeling that pharmacotherapy lacked options. Recently, alternative treatment options, such as psilocybin and LSD, have received attention [108,109,110,111,112]. Whilst these data should be treated cautiously, they represent an intriguing and promising area of exploration for future research, especially as they may provide fewer side effects than stimulant medication and focus on serotonin receptors, proving a potential alternative treatment route for those with comorbid SUD [113]. An in-depth discussion of potential psychedelic substances in the treatment of ADHD is beyond the scope of this discussion but warrants investigation. Concurrently, our qualitative data highlight that individuals desire a more patient-focused treatment. This resonates with data suggesting a complete absence of psychological therapy and limited follow-up once medication had been prescribed [114]. Finally, we highlight that 82.0% of the survey sample had sought treatment for their disorder through conventional means. We interpret this as support for our previous findings, inferring that NPS self-medication remains a “last resort” for most [20].

Whilst this study has strengths in its novelty, identifying a phenomenon that is hard to study experimentally, it is also not without limitations. Firstly, due to the nature of our forum-based convenience sample, we are unable to make any definitive comments or generalise our results to the larger population. Due to the non-experimental nature of the research, the evident sample bias, and the lack of analytical confirmation of the substances being used, caution must be taken in extrapolating findings, and further analytical research would be beneficial if undertaken. Additionally, criticisms of the survey methodology included individuals being unable to leave negative ratings (<0) and only being allowed one effectiveness rating per disorder, regardless of the amount of NPSs used to attempt to self-medicate. Due to this, we were unable to establish whether any specific NPSs were perceived as effective or non-effective. These changes should be considered for future research. Additionally, the current data only capture one moment in time, and, therefore, we are unable to provide a long-term perspective. It would be interesting to examine whether any improvements were consistently observable over time. It is, however, likely that a longitudinal study may uncover increased negative side effects as use becomes more prolonged. We also recognise the possibility of a social desirability bias within the survey sample [115] and acknowledge that many individuals within these forums are in favour of these novel substances remaining free from sanction or control. Any attempt to paint these novel substances in a positive light within the academic sphere could, in their eyes, push these substances further away from legislative control. These motivations may, either consciously or unconsciously, have impacted their responses. Despite these caveats, our study explores a unique phenomenon and demonstrates a clear willingness of some to self-medicate ADHD.

## 5. Conclusions

Overall, these findings provide novel insights into the phenomenon of self-medicating ADHD with NPSs. Our data support previous research, suggesting that individuals who have exhausted treatment options may be becoming more willing to explore self-medication. We recognise the need for more research to be undertaken to comprehensively understand individual motivations; however, we posit that the self-medication behaviours demonstrated here represent rational choices [62] and, therefore, that improving the access to and experiences of treatment for ADHD, as well as providing additional pharmacotherapy options, should reduce the incentives to self-medicate.

## Figures and Tables

**Table 1:** Table of disorders declared.

Disorder	*N*	(%)
Attention deficit disorder with hyperactivity (ADHD) (F90.0)	114	45.4
Attention deficit disorder without hyperactivity (ADD) (F98.8)	111	44.2
Attention deficit disorder with hyperactivity, inattentive (ADHD-PI) (F90.0)	6	2.4
Autism (F84.0)	3	1.2
Attention deficit disorder combined (ADHD-C) (F90.2)	2	0.8
Non-attentional disorders		
Major depressive disorder (F32.9)	5	2.0
Generalized anxiety disorder (F41.1)	3	1.2
Post-traumatic stress disorder (PTSD) (F43.1)	2	0.8
Cognitive communication deficit (R41.84)	2	0.8
Brain fog (unspecified) (R41.9)	1	0.4
Chronic fatigue (G93.32)	1	0.4
Narcolepsy (G47.419)	1	0.4

**Table 2:** Table of perceived effectiveness.

Question	Mean Score (±*SD*)
“On a scale of 1–100, how well do you feel the treatment worked?”	
Conventional treatment	55.5 (26.5)
NPS self-medication	71.3 (22.1) *
“On a scale of 1–100, how much did your symptoms improve?”	
Conventional treatment	51.4 (27.7)
NPS self-medication	69.0 (21.7) *
“On a scale of 1–100, how much did your quality of life (QOL) improve?”	
Conventional treatment	52.0 (30.5)
NPS self-medication	63.8 (27.3) *
Healthcare perceptions	
How competent do you view professional healthcare?	42.4 (24.7)
How well do you think you are supported by the medical healthcare and mental health system?	37.2 (28.0)
How do you rate your access to healthcare needs?	53.4 (30.2)

* Paired-sample *t*-tests showed a significant difference between all three questions (*p* < 0.001 = “Did it work”, “Did symptoms disappear”, “Did QoL increase?”).

**Table 3:** Most used NPSs to self-medicate ADHD.

Substance	Total (%)
4F-MPH (*4-fluoromethylphenidate*)	49.6%
2-FMA (*2-fluoromethamphetamine*)	46.0%
isopropylphenidate	20.4%
2-FA (*2-fluoroamphetamine*)	19.2%
3-FPM (*3-fluorophenmetrazine*)	17.2%
1P-LSD (*1-propanoyl-lysergic acid diethylamide*)	14.4%
NEP (*N-ethylpentedrone*)	12.4%
3-FA (*3-fluoroamphetamine*)	12.0%
etizolam	11.6%

## Data Availability

The data that support the findings of this study are available from the corresponding author upon reasonable request due to potential ethical issues.

## Supplementary Materials

The following supporting information can be downloaded at https://www.mdpi.com/article/10.3390/brainsci15030292/s1. S1: Survey copy. S2: Interview guide. S3: Codetree, S4. Qualitative themes.
