# Supplementary material for: The Use of Novel Stimulants in ADHD Self-Medication: A Mixed Methods Analysis
Source: Brain Sci. 2025 Mar 10;15(3):292. doi: 10.3390/brainsci15030292 (PMC11940814; doi:10.3390/brainsci15030292)
Supplement: Supplementary file 1 [file brainsci-15-00292-s001.zip › ADHD survey (S1).pdf]

Consent Form

INFORMATION SHEET FOR PARTICIPANTS

Ethical Clearance Reference Number:

Title of project

Self-rated effectiveness of novel psychoactive substances (NPS) in self-medicating attention deficit disorders (ADHD/ADD).

Invitation paragraph

I would like to invite you to participate in this PhD research project investigating perceived effectiveness of novel psychoactive substances (NPS) in self-medicating attention deficit disorders (ADHD/ADD). Before you decide whether you want to take part, it is important for you to understand why the research is being done and what your participation will involve. Please take time to read the following information carefully and discuss it with others if you wish. Ask me if there is anything that is not clear or if you would like more information.

What is the purpose of the project?

The purpose of the study is to assess self-medication with NPS. There is evidence to suggest that individuals may be self-medicating with NPS such as 2-FMA and 4F-MPH, however this is scarcely represented in the academic literature. We aim to examine the self-rated effectiveness (SRE) of novel psychoactive substances (NPS) for attention deficit disorders, compared to conventionally prescribed treatments. As a proponent of evidence-based drug policy, we hope that understanding how NPS are being used can help to positively influence drug policy.

Why have I been invited to take part?

You are being invited to participate in this study because we are interested to hear from the general public. If you are 18-years old, or older, understand English, and have used/are using a NPS to self-medicate an attentional deficit disorder, then you are eligible to participate in this study. To participate you do not require a formal attentional deficit disorder diagnosis.

Within this study novel psychoactive substances (NPS), also known as legal highs or research chemicals, are defined as either analogues of existing controlled drugs and pharmaceutical products, or newly synthesised chemicals created to mimic the actions and psychoactive effects of licensed medications and other controlled substances. These can be legal,

however may also include controlled substances as definitions of NPS vary between countries, reflecting differences in national legislation.

What will happen if I take part?

If you agree to take part you will complete a survey anonymously. The survey will take you approximately 15 minutes to complete. The survey will ask you questions about your gender, age, ethnicity, country of residence, medication you may be using and its effectiveness, NPS you may be using and its effectiveness and your perceptions of professional healthcare. The survey will also ask about the attentional deficit disorder you are suffering from and your mental health experiences. Examples of questions you may be asked include:

- Have you ever had, or do you currently have an attentional deficit disorder?
- Which attention deficit disorder did/do you have?
- Have you undertaken medical treatment for your disorder? If so, what type?
- How effective was the treatment offered for your attention deficit disorder?

Do I have to take part?

Participation is completely voluntary. You should only take part if you want to and choosing not to take part will not disadvantage you in anyway. If you choose to take part you will be asked to provide your consent. To do this you will be asked to indicate that you have read and understand the information provided and that you consent to your anonymous data being used for the purposes explained.

What are the possible risks of taking part?

You will be asked about your NPS and other substance use, any mood or anxiety disorder you may be suffering from and your perceptions of professional healthcare. Should you become distressed when participating you can exit the survey at any time. Should you experience any psychological discomfort we have provided support services listed at the bottom of this information sheet. Due to the nature of the research there is a risk of illegal disclosures or disclosures that could result in personal/professional reprisal if revealed to the wrong people. To mitigate these risks we have ensured full anonymity of the survey and will delete identifiable disclosures made accidently. To further mitigate this participants should be careful not to include any potentially identifying information in their responses. Examples of this could include names, age, location, details of where you work, names of colleagues, or any other references to your professional occupation.

If you feel you need support for any issues raised in this study please use the following support services:

• NHS: If you need help urgently for your mental health, but it's not an emergency, get help from NHS 111 online or call 111 • Mind: https://www.mind.org.uk/ or call 0208 215 2243

• Mental health: https://www.mentalhealth.org.uk/explore-mental-health/get-help

• Samaritans: Call 116 123

• ADDISS - National Attention Deficit Disorder Information and Support Service (UK): call 020 8952 2800

• HelpGuide.org – International help and information regarding ADHD: https://www.helpguide.org/home-pages/add-adhd.html

What are the possible benefits of taking part?

There are no direct benefits to you in taking part.

Data handling and confidentiality

This research is anonymous. This means that nobody, including the researchers, will be aware of your identity, and that nobody will be able to connect you to the answers you provide, even indirectly. Your answers will nevertheless be treated confidentially and the information you provide will not allow you to be identified in any research outputs/publications. Your data will be held securely in encrypted devices, at the end of the study the anonymous data will be deposited with King's Research Data Management System (DMS) under a Creative Commons Attribution licence (CC-BY). Any personal data collected independently of the anonymous survey as described elsewhere in this information sheet will be processed under the terms of UK data protection law (including the UK General Data Protection Regulation (UK GDPR) and the Data Protection Act 2018). If you would like more information about how your data will be processed under the terms of UK data protection laws please visit the link below:

https://www.kcl.ac.uk/research/support/research-ethics/kings-college-london-statement-on-use-of-personal-data-in-research

Data Protection Statement

If you would like more information about how your data will be processed under the terms of UK data protection laws please visit the link below:
https://www.kcl.ac.uk/research/support/research-ethics/kings-college-london-statement-on-use-of-personal-data-in-research

What if I change my mind about taking part?

You are free to withdraw at any point during completion of the survey, without having to give a reason by closing the browser window. Your answers will not be saved until you submit the survey. Withdrawing from the study will not affect you in any way. Once you submit the survey, it will no longer be possible to withdraw from the study because the data will be fully anonymous. Please do not include any personal identifiable information in your responses.

What will happen to the results of the project?

The results of the study will be summarised in a published dissertation. There is potential for the results to be published in a journal or presented at a conference.

Who should I contact for further information?

If you have any questions or require more information about this study, please contact me using the following contact details:

Tayler Holborn – PhD Addictions Research student

Email: tayler.j.holborn@kcl.ac.uk

King's College London - Addictions Department 4 Windsor Walk SE5 8BB

What if I have further questions, or if something goes wrong?

If this project has harmed you in any way or if you wish to make a complaint about the conduct of the project you can contact King's College London using the details below for further advice and information:

Supervisor: Paolo Deluca – Reader of Addictions research

Email: paolo.deluca@kcl.ac.uk

King's College London - Addictions Department 4 Windsor Walk SE5 8BB

Thank you for reading this information sheet and for considering taking part in this research.

Please confirm the following statements before beginning the survey:

- [ ] I confirm that I have read and understood the previous information sheet for the above project and agree to take part in the study. I have had the opportunity to consider the information and asked questions which have been answered to my satisfaction.
- [ ] I consent voluntarily to be a participant in this project and understand that I can refuse to take part and can withdraw from the project at any time, without having to give a reason. After submission of the survey, data will not be able to be withdrawn.
- [ ] I consent to the processing of my personal information for the purposes explained to me in the Information Sheet. I understand that such information will be handled under the terms of UK data protection law, including the UK General Data Protection Regulation (UK GDPR) and the Data Protection Act 2018.
- [ ] I understand that my information may be subject to review by responsible individuals from King's College for monitoring and audit purposes.
- [ ] I understand that confidentiality and anonymity will be maintained, and it will not be possible to identify me in any research outputs.
- [ ] I understand that the information I have submitted may be published as a report.

Please complete the follow CAPTCHA:

- [ ] I'm not a robot

reCAPTCHA
Privacy - Terms

Demographics

Q1: What is your gender?

- Male
- Female
- Non-binary / third gender
- Prefer not to say

Q2: What is your age?

Q3: What best describes your ethnic origin?

- American Indian/Native American or Alaska Native
- Asian (Indian, Pakistani, Bangladeshi, Chinese, any other Asian background)
- Black, African, African American or Black British
- Hispanic, Latino or Spanish origin
- Middle eastern or North African
- Native Hawaiian or Other Pacific Islander
- White or Caucasian
- Mixed two or more ethnic groups
- Other
- Prefer not to disclose

Q4: In which country do you currently reside?

Q5: Which of these best describes your personal income last year?

- $0 (£0)
- $1 to $9,999 (£1 - £8300)
- $10,000 to $24,999 (£8310 - £20,780)
- $25,000 to 49,999 (£20,781 - £41,560)
- $50,000 to 74,999 (£41,561 - £62,330)
- $75,000 to 99,999 (£62,331 - £83,110)

- $100,000 to 149,999 (£83,111 - £123,885)
- $150,000 and greater (£123,886 +)
- Prefer not to answer

Q6: What is your highest level of education?

- Less than high school (Secondary school)
- High school graduate
- College (University)
- Vocational degree or similar
- Graduate or professional degree (MA, MSc, MRes, MS, MBA, PhD, JD, MD, DDS etc.)
- Prefer not to say

Drug use history

Q7: Which substances have you used, or are currently using? Tick all that apply.

- Alcohol
- Nicotine
- Cannabis
- MDMA
- Ketamine
- LSD
- Ayahuasca
- Magic mushrooms (Psilocybin)
- Stimulant NPS
- Hallucinogen NPS
- Cannabinoid NPS

NPS survey

The remainder of the survey will focus on your experiences self-medicating with Novel psychoactive substances (NPS). Please answer these questions as honestly and accurately as possible. Thank you.

Q8: Have you ever suffered, or do you currently suffer from an attention deficit disorder ?

- ○ No
- ○ Yes

Q9: Which attention deficit disorder do/did you suffer from? Please choose from below or use the following text box if not included. If you suffer from multiple, please pick one and you will be able to select another later in the survey.

- ○ Attention deficit disorder with hyperactivity (ADHD) (F90.0)
- ○ Attention deficit disorder without hyperactivity (ADD) (F98.8)
- ○ Other, include below:

Q9: If not listed above, please write your attention deficit disorder below.

[ ]

Q10: Was your disorder diagnosed by a medical professional?

- ○ No
- ○ Yes

Q11: Have you undertaken medical treatment for your disorder? If so, what type?

- ○ Medication
- ○ Therapy
- ○ Both
- ○ No
- ○ Other treatment, state on next page

If stated 'other' for the previous question, please include here. Otherwise leave blank.

[ ]

Q12A: On a scale of 1-100, how well do you feel the treatment worked?

	Didn't work at all							Worked significantly			
	0	10	20	30	40	50	60	70	80	90	100
Do you feel the treatment worked?											[ ]

Q12B: On a scale of 1-100, how much did your symptoms improve?

	No improvement									Completely disappeared	
	0	10	20	30	40	50	60	70	80	90	100
Did symptoms disappear?											

Q12C: On a scale of 1-100, how much did your quality of life (QOL) improve?

	Not at all									Improved significantly	
	0	10	20	30	40	50	60	70	80	90	100
How much did QOL improve?											

Q13: Have you ever used a novel psychoactive substance (NPS) in an attempt to self-medicate your disorder?

- ○ No
- ○ Yes

Q14: Which novel psychoactive substance(s) (NPS) did you use to treat your previously stated attention deficit disorder?

- ☐ Pyrazolam
- ☐ Etizolam
- ☐ Diclazepam
- ☐ Flualprazolam
- ☐ 2-FA
- ☐ 3-FA
- ☐ N-Ethylpentedrone (NEP)
- ☐ 4-FA
- ☐ 3-FMA
- ☐ 3-FPM
- ☐ 2-FMA
- ☐ 4F-MPH
- ☐ Isopropylphenidate
- ☐ 3-MeO-PCP
- ☐ Ketamine
- ☐ Deschloroketamine
- ☐ 2F-DCK

- ☐ 3-MeO-PCE
- ☐ Methoxetamine
- ☐ DXM
- ☐ O-PCE
- ☐ 2-BDCK
- ☐ 3-HO-PCE
- ☐ 4-AcO-DMT
- ☐ 1P-LSD
- ☐ 1CP-LSD
- ☐ O-DSMT
- ☐ U-47700

Q14: If your substance(s) is/are not included above please write in the following text box. Please avoid slang terms.

Q15A: On a scale of 1-100, how well did self-medicating with a NPS work? If multiple NPS were used for one disorder please give an overall evaluation.

	Didn't work at all									Worked well	
	0	10	20	30	40	50	60	70	80	90	100
Do you feel self-medication worked?											

Q15B: On a scale of 1-100, how much did your symptoms disappear?

	Didn't disappear									Completely disappeared	
	0	10	20	30	40	50	60	70	80	90	100
Did symptoms disappear?											

Q15C: On a scale of 1-100, how much did your quality of life (QOL) improve?

	Not at all									Improved significantly	
	0	10	20	30	40	50	60	70	80	90	100
How much did QOL improve?											

Q16: Do you suffer from another attentional disorder aside from the one just declared, for which you have self-medicated with NPS?

- No
- Yes

Q9: Which other attention deficit disorder do/did you suffer from? Please choose from below or use the following text box if not included.

- Attention deficit disorder with hyperactivity (ADHD) (F90.0)
- Attention deficit disorder without hyperactivity (ADD) (98.8)
- Other, include below:

Q9: If not listed above, please write your attention deficit disorder below.

Q10: Was your disorder diagnosed by a medical professional?

- No
- Yes

Q11: Have you undertaken medical treatment for your disorder? If so, what type?

- Medication
- Therapy
- Both
- No
- Other, include on next page

If stated 'other' for the previous question, please include here. Otherwise leave blank.

Q12A: On a scale of 1-100, how well do you feel the treatment worked?

	Didn't work at all							Worked significantly			
	0	10	20	30	40	50	60	70	80	90	100
Do you feel the treatment worked?											

Q12B: On a scale of 1-100, how much did your symptoms improve?

	No improvement						Completely disappeared				
	0	10	20	30	40	50	60	70	80	90	100
Did symptoms disappear?											

Q12C: On a scale of 1-100, how much did your quality of life (QOL) improve?

	Not at all						Improved significantly				
	0	10	20	30	40	50	60	70	80	90	100
How much did QOL improve?											

Q14: Which novel psychoactive substance(s) (NPS) did you use to treat your previously stated attention deficit disorder? Pick all used.

- ☐ Pyrazolam
- ☐ Etizolam
- ☐ Diclazepam
- ☐ Flualprazolam
- ☐ 3-FPM
- ☐ 2-FA
- ☐ 3-FA
- ☐ N-Ethylpentadrone (NEP)
- ☐ 4-FA
- ☐ 3-FMA
- ☐ 2-FMA
- ☐ 4F-MPH
- ☐ Isopropylphenidate
- ☐ 3-MeO-PCP
- ☐ Ketamine
- ☐ Deschloroketamine
- ☐ 2F-DCK
- ☐ 3-MeO-PCE
- ☐ Methoxetamine
- ☐ DXM
- ☐ O-PCE
- ☐ 2-BDCK
- ☐ 3-HO-PCE
- ☐ 4-AcO-DMT

- ☐ 1P-LSD
- ☐ 1CP-LSD
- ☐ O-DSMT
- ☐ U-47700

Q14: If your substance(s) is/are not included above please write in the following text box. Please avoid slang terms.

Q15A: On a scale of 1-100, how well did self-medicating with a NPS work? If multiple NPS were used please give an overall evaluation.

	Didn't work at all								Worked well		
	0	10	20	30	40	50	60	70	80	90	100
Do you feel self-medication worked?											

Q15B: On a scale of 1-100, how much did your symptoms disappear?

	Didn't disappear							Completely disappeared			
	0	10	20	30	40	50	60	70	80	90	100
Did symptoms disappear?											

Q15C: On a scale of 1-100, how much did your quality of life (QOL) improve?

	Not at all							Improved significantly			
	0	10	20	30	40	50	60	70	80	90	100
How much did QOL improve?											

Q17: Which came first, your NPS use or diagnosis of attentional deficit disorder?

- ○ Medical diagnosis
- ○ NPS use

If undiagnosed, have you experienced difficulty obtaining a diagnosis as an adult?

- ○ Yes
- ○ No

Block 4

Q18: Overall, out of 100, how competent do you view professional healthcare in treating attention deficit disorders?

	Not competent							Very competent				
	0	10	20	30	40	50	60	70	80	90	100	
Competence of professional healthcare												

Q19: Out of 100, how well do you think you are supported by the medical healthcare and mental health system?

	Not at all							Very supported				
	0	10	20	30	40	50	60	70	80	90	100	
How well supported are you?												

Q20: Out of 100, how do you rate your access to healthcare needs?

	No access							Good access				
	0	10	20	30	40	50	60	70	80	90	100	
How accessible is professional healthcare to you?												

Q21

Q19: If you would like to be included in future psychoactive substance research, please follow the link below. No identifiable data will be linked to your responses in this survey.

https://qualtrics.kcl.ac.uk/jfe/form/SV_5ceTcBcBcpa5ddk

Powered by Qualtrics
