# Supplementary material for: The Use of Novel Stimulants in ADHD Self-Medication: A Mixed Methods Analysis
Source: Brain Sci. 2025 Mar 10;15(3):292. doi: 10.3390/brainsci15030292 (PMC11940814; doi:10.3390/brainsci15030292)
Supplement: Supplementary file 1 [file brainsci-15-00292-s001.zip › Codetree (S3).pdf]

NPS Framework analysis

Codes

Name	Description
Countries	
Bulgaria	
Germany	
Greece	
Netherlands	
Dutch culture	
UK	
NHS	
Disorders	
ADHD	
ADHD inattentive	

Name	Description
Adult ADHD	
Hyperactivity	
School learning issues	
Anhedonia	
Anxiety	
Social anxiety	
Autism	
Comorbid disorder	
Depression	
Insomnia	
Narcolepsy	
PTSD	
Good quotes	
Healthcare interaction	

Name	Description
Access	
Complex process	
Cost	
Insurance	
Long wait	
ADHD diagnosis	
Not a disorder view	
Healthcare perceptions	
Addiction	
Competence	
Dismissive psychiatrist	
Finding a good psychiatrist	
Generational change	
Humility	

Name	Description
Lack of knowledge	
Patient centred	
Psychiatrist cooperation	
Stigma	
Therapy	
NHS	
Novel treatments	
Legal ketamine treatment	
MDMA therapy	
Psychedelic retreat	
Openness	
Medical surgery issue	
Individuals behaviour and views	
Drug knowledge	

Name	Description
Drug culture	
Harm reduction	
Psychonaut	
Family	
Conservative parents	
Early drug use	
Friends and family support	
Information gathering	
Academic research	
Pharmacology	
Online information	
PsychonautWiki	
Reddit	
Life	

Name	Description
Being old	
Childhood	
Politically involved	
Studying and academia	
Education	
School	
Work	
Distracted	
Job	
Mental Health	
Suicidal ideation	
Trauma	
Personal experiences and views	
Neurodivergence	

Name	Description
Personal view	
Personality	
Addictive personality	
Risk taker personality	
Recreational drug use	
Bad trip	
Dark web	
Drinking	
Polysubstance use	
NPS substance	
Benzodiazepines	
Bromazolam	
Clonazolam	
Flualprazolam	

Name	Description
Flubromazolam	
Borax	
6APB	
Dissociatives	
2FDCK	
Deschloroketamine	
DMXE	
DXM	
FXE	
Modafanil	
Nicotine	
Socially accepted	
Opioids	
Psychedelics	

Name	Description
1PLSD	
2CB	
LSA	
Stimulants	
3FPM	
Amphetamines	
2FMA	
3FMA	
4-FMA	
4FMA	
BZP	
Cathinones	
2MMC	
Mephedrone	

Name	Description
Phenidates	
4F-MPH	
Isopropylphenidate	
Prescription medication	
Experiences	
Access	
Ineffective	
IR vs XR	
Lack of options	
Medication shortages	
Stimulant averse	
Tapering	
Medication types	
Adderall	

Name	Description
Antidepressants	
Atomoxetine	
Elvanse	
Gabapentin	
Lyrica	
Ritalin	
SSRIs	
Side effects	
Emotional blunting	
Sweating	
Self-medication	
Dosages	
Manic	
Effects	

Name	Description
Concentration	
Euphoria	
Focus	
Functional	
Motivation	
Legality	
Blanket ban	
Change in law	
Mindset	
Discipline	
Last resort	
Responsibilty	
'Soul' therapy	
Motivations of use	

Name	Description
Age restriction	
Cheap	
Chronic issues	
Control	
Curative	
Different brain chemistry	
Discrete	
Feeling stuck	
Introspection	
New perspective	
Privacy	
Slow onset prescription	
Supplement medication	
NPS access	

Name	Description
Dealers	
Easy to order online	
Moving country issues	
No ID required	
Vendor trust	
NPS Side effects	
Amnesia	
Blackout	
Body pain	
Cognitive function	
Compulsivity	
Exhaustion	
Fluorinated poisoning	
Hyperfocus	

Name	Description
Lymph node	
Memory	
Paranoia	
Rebound anxiety	
Tinnitus	
Tolerance	
Withdrawals	
Other	
Abstinence	
Drug dealers	
Quality	
Counterfeit seeds	
Drug Testing	
Mislabelling	

Name	Description
Unsure what substance was	
Reasons for stopping	
Loss of interest	
Sobriety	
Risks	
No oversight	
Running out	
Safety	
Use profile	
Daily use	
Duration of use	
Ketamine research	
ROA	
Weight loss	
