# Supplementary material for: The Use of Novel Stimulants in ADHD Self-Medication: A Mixed Methods Analysis
Source: Brain Sci. 2025 Mar 10;15(3):292. doi: 10.3390/brainsci15030292 (PMC11940814; doi:10.3390/brainsci15030292)
Supplement: Supplementary file 1 [file brainsci-15-00292-s001.zip › Interview template (S2).pdf]

Semi-structured interview template

Participants ID:	Date:	Start:	End:
Email:	M. Teams/Telephone: Teams		
Gender:	Disorder being self-medicated:		

Informed consent

Hello, _____. How are you today? Thank you for attending this interview.

Let me introduce myself. My name is Tayler. I am a researcher at King's College London, and I am investigating how individuals may be using novel psychoactive substances (NPS) to self-medicate a variety of disorders and I am interested your experiences, as well as your perceptions of conventional healthcare.

Before we start, I would like to remind you that we will be talking about topics that can be sensitive for some individuals and so we recommended you are alone and using headphones to protect your privacy. You can also choose to turn off your camera at any point should you want. If you happen to feel uncomfortable during the interview or do not want to answer any questions, then please let me know.

This interview will take from 30 minutes to 1 hour.

From this point forward, our conversation will be recorded. Is that OK?

**Preliminary questions**
Have you read and understood the Participant information sheet?<br>Do you have any questions about the participation in this study?<br>Can you confirm that you would like to take part in an interview about self-medication with novel psychoactive substances (NPS)?<br>Do you consent to the recording of this interview?
**Rapport and history of NPS use (10 mins)**
OK, ________. Maybe we could start with you just telling me when you first used, or heard about research chemicals/legal highs/ novel psychoactive substances?<br>- How old were you?<br>- Which substance(s) did you use?<br>- Where did you obtain the NPS from?<br>- Was your use recreational or an attempt to self-medicate?

**NPS use (10 mins)**
What types of NPS do you use now to self-medicate??<br>How do you usually consume them? (e.g. smoke them, swallow pills)<br>Where do you get them from? (e.g. headshop, online, friend, relative, dealer)<br>How often do you use them? And how long have you been using for? (e.g. every day)<br>What dose would you use?<br>How do you view the quality of the NPS substances you use?<br>How do you view the safety of the NPS substances you use? (fluorinated analogues)<br>Do ever test your substance or practice any harm reduction?

Self-medication (10 mins)
Which disorder have you attempted to self-medicate?<br>Tell me about why you choose/chose to self-medicate with NPS<br>What are some of the positive effects that self-medicating has had on you?<br>What are some of the negative effects that self-medicating has had on you?<br>Why did you choose to self-medicate with an NPS over prescription medication from a professional?<br>In what ways does self-mediating with an NPS differ from using prescription medication?<br>Have you received a diagnosis?<br>In your eyes, has self-medication been successful?<br><br>Does the wide choice of NPS influence your decision to choose them over a normal prescription substance, if so, why?<br>How important is the role of an online community/discussion platform in your use of NPS?<br>Do you enjoy taking an interest in novel pharmacology/drug science?<br>Do you feel current academic substance research affects your choice of NPS to self-medicate with, if so, how?<br>Overall, how has taking NPS impacted your disorder?<br>Do you feel they have affected your mental health, and if so, how?<br>Do you feel they have affected your physical health, and if so, how? Have you noticed any side effects? (e.g. heart issues from long term stimulant use?)

Perceptions of professional healthcare (10 mins)
I'm just going to ask a few questions about your perceptions of professional healthcare.<br>How competent do you feel professional healthcare is in treating your disorder?<br>Do they have sufficient tools to help you overcome your disorder, if not, what is lacking?<br>Do you feel as though you have sufficient access to your healthcare needs?<br>In what ways have your experiences of professional healthcare influenced your decisions to use NPS?

Conclusion
Is there anything else you would like to add regarding your experiences self-medicating with novel psychoactive substances (NPS)?<br>Would you like to receive a link to this study via email when the research is published?<br>Do you have any questions for me?

So according to our ethical procedures, you have two weeks to decide if I am allowed to use your interview in this study.

Therefore, if by _____ you haven't asked me to delete your interview, then it will be used in the study. Do you understand?

Thank you again for taking part in this study. Bye!

Stop recording. Leave the meeting. Inform the time the interview ended, on page 1.
