# Supplementary material for: The Use of Novel Stimulants in ADHD Self-Medication: A Mixed Methods Analysis
Source: Brain Sci. 2025 Mar 10;15(3):292. doi: 10.3390/brainsci15030292 (PMC11940814; doi:10.3390/brainsci15030292)
Supplement: Supplementary file 1 [file brainsci-15-00292-s001.zip › Qualitative quotes (S4).pdf]

**Theme 1 – Initiating NPS use**	***Quotes***
Struggling academically, socially, or issues with self-perception (weight loss)	*"In university or at school for exams, like my mind would feel by itself [even] without any diagnosis that I need amphetamines to pass the exams, because if not then I will be just very, very slow in completing them."*
	"Yeah, so I think it's been a while that I've been suspecting ADHD because I've read testimonies online, which I felt were close to what I was feeling. And I also checked Wikipedia, […] which did correspond to my situation."
	"I started using amphetamines, actually not in order to self-medicate, but at that point in order to lose my weight."
	"So I started searching for a research chemical for ADHD and I found several posts talking about 2fma so I tried that"
	"in university now, I was like it's getting a little harder so or like just if I would like go longer breaks that studying so it was really hard to get back into studying stuff like that so I was just went on like reddit was like "oh what's like a clean like dopamine reuptake inhibitor like easy focus"? and there was like a couple, there was a methylphenidate like derivative I think, but I was just more attracted to like the fluorinated methamphetamines so 2FMA…"
	"At the time when I was a student and when I was feeling that this is becoming to be a problem for me, not following my peers and not understanding what the teacher is talking about in class, it was a problem, and motivation to finish things and many things associated with ADHD. I read about it on the internet and I kind of self-diagnosed and started to self-treat"
Internet exposure	*"First time I read about Modafinil on Reddit. I'm not a big Reddit user […] but I read about Modafinil there for the first time. And then I read about 2FMA there too about people who are using it, and they worked for them and etc. And I risked by trying it…"*
Background	*"I'm originally from Russia and it's only very recently that they focus on one neurodivergent diagnosis. No one ever diagnosed me, no one ever treated me, no one ever really wanted to focus on that."*
	"With the background of trauma, abandonment… trauma when I was eight, I was always growing like a rebel kid and always ultra independent as well."
Academic interest	*"I have always been very intent on studying and researching everything that I intend to take. I'm very interested in drugs and I'm studying drugs academically, but I was also trying to figure out how to use everything in as minimally harmful a way as possible, while still like trying to self-medicate"*
	"So, before I tried, I did pretty much of research on it and I keep on going and because this kind of chemistry is my hobby."
	"So, I have seen the paper, for example, for isopropylphenidate. I mean, the one stating it's actually better, and I mean it has fewer side effects and better longer half-life and it's better than methylphenidate…"
	"Neuroscience is like the first thing I really, really enjoyed. It was the first thing that sort of gave my life any meaning or gave me a reason to wake up in the morning."

Restrictions	*"I had already been using some substances before then, but was looking for stuff that did not have age restrictions associated with it as I was not 21, or 18"*
	"I was having trouble obtaining prescriptions that I found useful to me for some various mental health stuff I had going on at the time. So decided to look around elsewhere and found a pretty accessible market in the research chemical spaces"
Stop-gap or supplementation	*"I already had an issue with the dosage of it [Ritalin] because for some reason, I have a high natural tolerance to where the maximum legal prescription where I live in France is 80 milligrams, which is not high enough for me from the beginning…"*
	"I feel like it is less potent than Ritalin normally is, because I snorted both, […] and also another thing with Ritalin that I get, I can only get delayed release here or extended release, which also I don't notice any effect…"
	"The problem was that even when I first got my Ritalin prescription for the first time in my life. I got diagnosed when I was 19. And I already had an issue with the dosage of it because for some reason, I have a high natural tolerance to where the maximum legal prescription where I live in France is 80 milligrams, which is not high enough for me from the beginning."
	"It [self-medication] was actually to find something which acts much faster. It's not the basic amphetamine, because it's way too expensive in Germany. Yeah, and then I found the 3FPM."
	"For ADHD was looking at novel stimulants. At first, I didn't go too crazy with those, I was looking at some more mild options and also analogues of like prescription stimulants, like four-fluoromethylphenidate comes to mind, four-methylphenidate, 4- METMP, I don't quite remember what that one stands for, isopropylphenidate. And then eventually I started looking at some stronger alternatives, like cathinones, some other substituted amphetamines."
	"the way I usually use it is I supplement my normal Ritalin doses…"
	"I have been using Isopropylphenidate for some time. 2FMA was also a good, I mean, it's also good, but I find phenidates to be more effective, at least for me."
	" I would, for example, use half of my, like 20 milligrams of Ritalin in the morning, and then for the rest of the day, use the 4FMPH until like in the afternoon, I use another 40 milligrams of Ritalin."
	"once I run out of Ritalin, there is obviously, if I don't have anything to substitute it with, there is obviously a week or two week period where I kind of I guess it's like tolerance break sort of but worse because it just quit in cold Turkey so it worsens the symptoms of ADHD a lot...

	"the one I get relatively regularly, or used to get, I haven't ordered it for a few months now. But every couple of months I would get for 4FMPH, which is the Ritalin "replacement"."
Dealers	*"And the reason I was interested in research chemicals was actually […], I didn't want to deal with dealers...."*
Access	*"Yes, the access is very limited. And then […] for Berlin, for example, or Hamburg, I am like 300 km from there, so that's a three-hour ride, no matter with what, if by car or on the train or whatever, it's too far, so yeah, it's pretty complicated."*
	At that point I would just get: "We are all overloaded with the work. We cannot take you in and you have to ask somewhere else". And frankly, I don't have time to travel 200 kilometers to visit a psychologist.
	"I have a lot of friends […] trying to get diagnosed in the UK in general and a lot of them are on like year-long waiting lists for the screening"
	"We had […] only one hospital for all Barcelona that has ADHD department and psychiatrists specialized in that. You have two years of the waiting list for the first consultation…"
	"First I went to the GP and the process was really complicated and it was not clear to me that I would get an appointment and how long it would take and then a bit later I found out about research chemicals and so that made me want to try."
	"when I was looking at specialists either they had stopped working on this, or the waiting list was full, or they weren't covered by my health insurance, so it was hard to find"
	"the main problem is I don't live in a big city. It's a rather small city with about 50000 citizens. So the care or the quality of this care is, let's say, quite low."
	"To get proper ADHD medication in Germany is generally a big problem because, yes, it's amphetamine and […] they are anxious that we just sell it on the black market or whatever. And so they are very, very saving with the prescription for amphetamines."
	"There are plenty of psychologists in this city, but they are all busy and they Just told me go away. I don't have any free capacity for you."
Cost/insurance	*"she prescribed it but it was so expensive and at that point my social security was not paying for it so it was 89 euros and I thought oh my god methamphetamine in Berlin cost 70, in Barcelona 50 euros and this is […] not even a gram…"*
	"I bought 3fpm, 1 gram for... I don't know, like 20 euros? And if I get a prescription amphetamine sulfate from my psychiatrist, I pay for 10 milligrams tablets, I pay like 80 euros and there are 30 pills so this ratio is just terrible…"
	"In Greece I don't have access to the health care services because I'm not paying any kind of healthcare insurance."
	Yeah and if I don't have money like me now I cannot pay for it [prescription]. So there's a financial element as well the costs and everything.
	"I tried to find a legal job that pays normally and to have insurance and everything but guess what? When you are 60, nope, you are out of luck entirely. [..] I do what I have to do to survive."
**Theme 2: Finding the "right" psychiatrist**	*"I think I might have just gotten lucky with the psychiatrist I have because he kind of accepted it at face value when I told him I had those suspicions […] that I might have ADHD, and from then on the process of getting diagnosed was actually surprisingly easy."*

	"It's just that, I mean, I found a good psychiatrist with whom I could speak with this without any concerns and we figured out the best course of action."
	"I had the luck that I found my psychiatrist which is able to prescribe me some proper medication, amphetamines..."
	"It was not too hard for me, but this was really because I happened to meet the right psychiatrist. Someone who understood that I may have done some experiments and probably know that stimulants are actually helping me quite a lot."
**Theme 3: Self-medicating**	
Dosage	*"I usually take about 10 to 15 milligrams in the morning and then that's it for the day. It does help for motivation and it's also easier for me to keep focus on something for a longer time."*
	"I'm using 2FMA for like Monday, Wednesday and maybe Friday, a strong dose that's like 45 milligrams, that's one and a half pills once around 11 a.m."
	"I would take like 50 milligrams at most. I mean, throughout the day once or twice, three times at most, but I avoided using this in the evening because then you wouldn't sleep well"
	"With 3FPM, actually I only eat it so I don't snort it, I drop like 30/40 milligrams which is corresponding something like 15/20mg of Amphetamine, maybe less even."
	"So it was usually like a 15, like half of a 30. So like 15 milligrams, sometimes even a quarter. If I like just wanted like a small boost, but yeah, usually like 15 milligrams to 2FMA was like the go to."
	"I mean I got to control my dose a lot more, […] when I was young it was just the physician or whatever would be like: does it feel like it's working? No? okay will up your dose a little bit.."
	"I don't exactly remember what dosages I was taking. I was taking pretty high dosages every day for like a little bit when I was beginning with some of these novel substances, but towards the end I was taking more moderate doses"
	"I found several posts online talking about 2FMA, so I tried that and I think I made a big mistake of not trying it orally from the beginning but from sniffing a small line, and at the beginning it was controllable but even from small amounts it was impossible to sleep, […] and yeah it's also became compulsive...."
	"The key is in the like, for instance, the dose. And I mean, you have to be precise and you need to have discipline."
Privacy	*"I just was not super interested in like going to see a therapist, going to see a doctor to get a prescription, which my parents are gonna have to, you know, put through to the insurance and they're gonna see all this stuff."*
Legality	*"Since then, I've used 3FMA and 4FMA, and 4FMA is a bit different. Nowadays 2FMA became very difficult to find because it became illegal in most countries."*

	"I made this decision because I saw I had a problem. I saw there is a solution, which obviously worked. I made this decision, but I didn't even tell my parents or anyone else because what can they say… break the law to self-medicate? it doesn't sound good but it actually helped me a lot and it was a right decision in the end."
Comorbid disorder self-medication	*"Barring the last two or three days I've been taking about 20 milligrams of 2CB, three times a day. So I, um, that's 20 milligrams as like the total amount, and I would take like five to seven milligrams three times to redose."*
	"I am also aware that using amphetamine on daily basis is not so exactly a nice thing, which brings me to a second point and this is it's not medically confirmed but it's probably the main issue of ADHD from my point of view and this is PTSD which I'm trying to also to solve with research chemicals."
	"I remember one time I, my first experience with Clonazolam, I got a bottle, a dropper bottle of the stuff and did a dose and then came to like a month later and didn't really remember much."
	"I tried to DIY ketamne treatment on myself when I came off SSRIs, but I found that NMDA receptor antagonists of any kind are extremely harmful to my body somehow..."
	"For depression, I looked at a lot of different things. I was interested in dissociatives, some different arylcyclohexylamines to mimic ketamine, which I had some interest in for antidepressive effects, as well as like psychedelics, similar interest there, was looking for something to help as a more one-off thing"
**Access**	*"So I think for me, so I used to sort of obtain my psychoactive substances through just a normal street dealer or through, you know, plants like LSA/LSH, but sort of when I started going on the dark web, it was a lot easier to obtain things that are a bit more rare and difficult to find, though, you know, the only thing I've really been buying in substantial amounts is 2CB"*
**Theme 4: Side effects**	*"In the past like I had been put on Ritalin and I found that to be like absolutely full of side effects whereas like 2fma was just a very clean just like not a lot of side effects."*
	"I don't use it [3-FPM] because I am afraid of the side effects, so actually I don't use them on the regular basis, I use them mostly if I know the start of the day will be hard."
	"Before going for a trip my one lymphatic node […] got super inflated like it was like painfully inflamed."
	"The sleep, the problem with sleep was insane. And also I started feeling the pain in my body."
	"I should say several years I haven't seen any bad side effects."
	"with this 4fmph, I had first couple of uses, I had pretty bad come down, it was like someone put my battery out and it was not huge amount, it was like 15 milligrams, […] it was really not pleasant."
	"I didn't experience any side effects […]. I am slightly anxious when I take it because I have still in my head possible poisoning with some other fluorinated amphetamines…"
**Theme 5: Healthcare perceptions**	*"I was thinking that that psychiatrist... she's just an idiot and that I know about ADHD much more than she does."*
	"I think overall the mental health care here does not know nearly as much about these conditions and these drugs as they are suggesting that they do"

	"It is a lot of just: a patient comes into the office, and you throw different medications at them until they stop complaining. And I think that that's not really the most responsible way to approach things, especially when a lot of these medications […] have a lot more side effects than is let on"
	"The overall attitude towards ADHD [in Bulgaria] is still kind of something coming from The West, which is actually not an illness, but something normal, which happens to a lot of children, and it's not something that should be treated."
	"I think the EU pushed us to have this [ADHD] diagnosis in place. But the people themselves, they still don't consider this an illness. It's something normal that most kids have to deal with."
	"Conventional therapy in Germany is useless for this kind of situation and I feel in my internal self I feel if I don't do something [...] then my life will be probably no longer be very long..."
	"I think the French healthcare itself isn't too bad for example I am getting the Ritalin for free or it's completely reimbursed"
	"I've had a lot of bad experiences with doctors in general, so I'm always a little suspicious of them. So it didn't really feel like there was any like method or any, I don't know, any expertise being applied."
	"They're kind of afraid of prescribing that kind of things. I had huge problems being prescribed modafinil even, which is a very mild stimulant."
	"I think they're afraid to prescribe stimulants for everyone. They're trying to avoid that. They don't know what they are actually doing."
	"the biggest issue I've had with a lot of them is that a lot of the time it does feel like you're just talking to a robot rather than a person..."
Theme 6: Reflections	*"I wish I had discovered them way earlier, because I lost so many jobs, so many lost opportunities, so many problems, so much suffering. For me it was hell on earth to do anything. […] It's not like I don't want to work. It's not like I'm not good at my work… Nobody was believing me."*
	"It's [self-medication] more like in your hands so you feel more like it's your responsibility to do it properly."
	"If I could, instead of supplementing the prescription I have with NPS, […] if I just got a better prescription than for sure, I would take it [instead]."
	"I feel like it's a lot harder to probably self-medicate yourself than use an extended release pill that you take once a day, So I've not really sought alternative."
	"I didn't want to be a criminal. I wanted to medicate myself."
	"I have a little bit of stress, you know, because at some point when I run out of 2FMA I have to replace it with something and right now I don't have a good alternative..."
	"If you have like a healthy social circle and like things to look forward to, […] it's much more attainable to do it [self-medication] responsibly, have people catch you on your downfalls, and like it's [self-medication] definitely net positive, at least in my experience, definitely net positive."-
	"I wish that there was more cooperation between the doctor and the patient to determine a good medication regimen for them. Like, I did not appreciate getting put on medications that I didn't find too helpful, but had a lot of side effects"
	"the alternative [to self-medication] is being completely dysfunctional and suicidal... I feel like all of my friends and family support my use of 2CB because they see I'm a lot more functional and a lot more capable of doing stuff and nobody really wants to deal with me when I'm just I'm completely inconsolable."

	*"What would **not** be a reason why I stopped taking them are that I think that prescription medications are inherently better. I don't. I don't have any more trust in prescription medications than I do in the NPS's on their own."*
	"I want to point out I never use it for just to get high or something like this, that I don't want to do... Not to get high, not to get hallucinate, but to try to heal myself a bit..."
	"I did not want to break the law. though principally I don't think that the law is good in general nothing good comes out of it but I never wanted to break the law and I mean I thought that these were more legal than they actually were"
	"Having had hyperactive friends who didn't finish high school, I kind of think if I did not start this self-treatment, legal or not, I would have been, I would have had the same fate."
	"It may be logical that, I mean, if you notice something is not right, you self-diagnose. I mean, there are only a few experts in this field who are mostly involved with ADHD and at diagnosing it, most even the psychiatrists, I mean, most of the psychiatrists aren't dealing with it."
	"over the last few months, I've been testing a theory that, you know, all of that use was making things worse for me, so I got off of everything very slowly, Prescription and NPS, and settled on these two prescriptions that I still have and have been making other lifestyle changes and overall things are better now than when I was using all of those."
	"a lot of people around me were not thrilled with me taking a bunch of research chemicals every day, but it's a little bit more socially acceptable to be on the prescriptions."
Openness	*"When I go to a doctor I'm going for a specific reason. either I want my prescription or whatever. I'm not going to tell them what I'm doing."*
	"I'm not open with my GP because I know he's not open to that and that's the big issue that because they don't know they won't say: "oh yeah that's a good idea or that's fine". Most doctors will not accept or, yeah, be open to people using NPS because they don't know anything about it…"
	"I told them stimulants have a positive effect on my alertness and my ability to finish tasks and I know this medication will work, but I haven't told them anything specifically about NPS and stuff."
	"I have had a lot of psychiatrists over the last couple of years, but I would occasionally try and very minimally talk about some of this stuff with them."
	"Mostly did not get good reactions out of that and found it more of a burden than anything to talk about this stuff with them."
